# Structural Ordering of Disordered Ligand-Binding Loops of Biotin Protein Ligase into Active Conformations as a Consequence of Dehydration

**DOI:** 10.1371/journal.pone.0009222

**Published:** 2010-02-15

**Authors:** Vibha Gupta, Rakesh K. Gupta, Garima Khare, Dinakar M. Salunke, Avadhesha Surolia, Anil K. Tyagi

**Affiliations:** 1 Department of Biochemistry, University of Delhi, New Delhi, India; 2 Department of Microbiology, University of Delhi, New Delhi, India; 3 National Institute of Immunology, New Delhi, India; University of Queensland, Australia

## Abstract

*Mycobacterium tuberculosis* (Mtb), a dreaded pathogen, has a unique cell envelope composed of high fatty acid content that plays a crucial role in its pathogenesis. Acetyl Coenzyme A Carboxylase (ACC), an important enzyme that catalyzes the first reaction of fatty acid biosynthesis, is biotinylated by biotin acetyl-CoA carboxylase ligase (BirA). The ligand-binding loops in all known apo BirAs to date are disordered and attain an ordered structure only after undergoing a conformational change upon ligand-binding. Here, we report that dehydration of Mtb-BirA crystals traps both the apo and active conformations in its asymmetric unit, and for the first time provides structural evidence of such transformation. Recombinant Mtb-BirA was crystallized at room temperature, and diffraction data was collected at 295 K as well as at 120 K. Transfer of crystals to paraffin and paratone-N oil (cryoprotectants) prior to flash-freezing induced lattice shrinkage and enhancement in the resolution of the X-ray diffraction data. Intriguingly, the crystal lattice rearrangement due to shrinkage in the dehydrated Mtb-BirA crystals ensued structural order of otherwise flexible ligand-binding loops L4 and L8 in apo BirA. In addition, crystal dehydration resulted in a shift of ∼3.5 Å in the flexible loop L6, a proline-rich loop unique to Mtb complex as well as around the L11 region. The shift in loop L11 in the C-terminal domain on dehydration emulates the action responsible for the complex formation with its protein ligand biotin carboxyl carrier protein (BCCP) domain of ACCA3. This is contrary to the involvement of loop L14 observed in *Pyrococcus horikoshii* BirA-BCCP complex. Another interesting feature that emerges from this dehydrated structure is that the two subunits A and B, though related by a noncrystallographic twofold symmetry, assemble into an asymmetric dimer representing the ligand-bound and ligand-free states of the protein, respectively. In-depth analyses of the sequence and the structure also provide answers to the reported lower affinities of Mtb-BirA toward ATP and biotin substrates. This dehydrated crystal structure not only provides key leads to the understanding of the structure/function relationships in the protein in the absence of any ligand-bound structure, but also demonstrates the merit of dehydration of crystals as an inimitable technique to have a glance at proteins in action.

## Introduction

Fatty acid biosynthesis is vital for the virulence and pathogenesis of *Mycobacterium tuberculosis* (Mtb), a deadly pathogen [1], [2]. The first committed step in lipid biosynthesis is the biotinylation of Acetyl Coenzyme A Carboxylase (ACC) mediated by biotin acetyl-CoA carboxylase ligase/biotin protein ligase (BirA) [3]–[5]. It has been shown that BirA is interchangeable between organisms [3], [6] suggesting conserved enzyme-substrate interactions. Yet, a recent biochemical study on Mtb-BirA has revealed significant differences in the ligand-binding properties of this enzyme compared to BirAs from various other organisms [7]. Therefore, on one hand, BirA appears to be an attractive target for the development of broad spectrum therapeutic agents against multiple infections, while on the other, it also appears to be ideal for the development of species-specific novel anti-infective agent.

Several crystal structures of both monofunctional and bifunctional BirAs [8], [9] from many genera have been determined either as apoenzyme or as complex with its ligands (Table 1) [10]–[14]. All the apo BirA crystal structures have revealed the presence of disordered flexible loops, which undergo a conformational transition upon biotin and biotinyl-5′-AMP binding. These loops are known to participate in either dimer interface or ligand-binding or both. The apo *Escherichia coli* (Ec) BirA has four-disordered loops - biotin binding loop:BBL, adenylate binding loop:ABL, dimer loop I:DLI and dimer loop II:DLII. Binding of ligands induces dimerization of EcBirA and structural ordering of these loops [12], [15]–[17]. However, *Pyrococcus horikoshii* (Ph) BirA exists as a dimer in both the liganded and unliganded forms [13] and the crystal structure of its apo form shows only one disordered loop (BBL) [10], [11]. Similarly, in the crystal structure of BirA from *M. tuberculosis* (Mtb-BirA), two such loops and a few residues at the N-terminal are missing (Ma and Wilmanns, PDB deposition 2cgh, 2006). Elucidation of molecular structure of these disordered loops may provide insights into species-specific behavior of Mtb-BirA towards its ligands.

**Table 1:** A comparative analysis of Mtb-BirA and other known bacterial BirA structures.

Organism (Function)	PDB	Identity (%)	N_aligned_	RMSD (Å)^‡^	Space group	V_M_	Solvent Content (%)	Mol per a.u.	Oligomeric state in solution	Ligand	Reference
*M. tuberculosis* (M)	3l2z	100	238	0.00	P2_1_2_1_2_1_	2.2	44	2	monomer	-	This study
*M. tuberculosis*	3l1a	100	238	0.82	P2_1_2_1_2_1_	1.7	28	2	monomer	-	This study
*M. tuberculosis*	2cgh	100	238	0.46	P2_1_2_1_2_1_	2.3	45.4	2	monomer	-	Unpublished
*E. coli* (B)	1bia	30.2	215	1.80	P4_3_2_1_2	2.8	55.6	1	monomer	-	[10]
*E. coli*	1bib	30.4	217	1.76	P4_3_2_1_2	2.8	55.5	1	dimer^†^	Biotin	[10]
*E. coli*	1hxd	30.1	219	1.71	C222_1_	2.9	57.5	2	dimer^†^	Biotin	[10]
*E. coli*	2ewn	29.5	220	1.72	P4_3_2_1_2	3.8	67.8	2	dimer	Biotinol-5′-AMP	[12]
*P. horikoshii* (M)	1wq7	27.2	206	1.55	P2_1_	2.1	38.1	2	dimer	-	[11]
*P. horikoshii*	1wpy	26.7	209	1.50	P2_1_	2.1	42.5	2	dimer	Biotin	[11]
*P. horikoshii*	2fyk	26.7	209	1.49	P2_1_	2.1	42.5	2	dimer	Biotin & ADP	Unpublished
*P. horikoshii*	2dto	26.7	209	1.52	P2_1_	2.1	42.5	2	dimer	Biotin & ATP	Unpublished
*P. horikoshii*	1wqw	26.7	209	1.53	P2_1_	2.1	42.5	2	dimer	Biotinyl- 5′-AMP	[11]
*P. horikoshii*	2 ejg	28.3	198	1.54	P2_1_	2.4	49.2	2	dimer	BCCP	[13]
*A. aeolicus* (M)	3fjp	21.4	196	1.52	P2_1_	2.4	48.2	2	monomer	-	[14]
*A. aeolicus*	3efs	21.4	196	1.52	P2_1_2_1_2_1_	2.3	45.5	2	monomer	Biotin & ATP	[14]
*M. jannaschii* (M)	2ej9	23.8	238	1.70	C2	2.8	56.1	1	monomer	Biotin	Unpublished

M: Monofunctional biotinylation activity; B: Bifunctional biotinylation and repressor activity; ^‡^The Cα superposition was performed between chain A of hMtb-BirA (3l2z) and chain A of other PDBs; V_M_: Mathews Coefficient; ^†^Weak dimer.

As reported earlier, the transfer of Mtb-BirA crystals to paraffin and paratone-N oil (cryoprotectants) and subsequent flash freezing resulted in the dehydration of the crystals and significantly reduced their solvent content [18]. Though the crystals diffracted at 2.69 Å, not better than the 1.8 Å data available in PDB for Mtb-BirA (2cgh), the most fortunate effect of the dehydration in our case turned out to be tracing of the structure of otherwise disordered ligand-binding loops absent in 2cgh and other apo BirAs. Involvement of these mobile loops in the biotinylation reaction is indispensable and hence the knowledge of their structure is critical for new leads into protein-ligand interactions. The conditions used in this study for the crystallization of Mtb-BirA protein were different from the one reported in 2cgh PDB entry. Hence, to eliminate the effects arising out of these differences, the Mtb-BirA structure (2.8 Å) was also determined with normal hydration for the purpose of comparison.

The dehydrated crystal structure of apo Mtb-BirA reported here provides the active structure of the disordered loops involved in the ligand-binding and functioning of BirA. Since in a dehydrated crystal, protein molecules are more densely packed leading to enhanced intermolecular contacts amongst themselves, dehydration emerges as a simple approach for restricting the conformational flexibility of the loop regions. Furthermore, partial reduction in the crystal's bulk solvent results in entrapping a state, where two protein subunits related by a non-crystallographic twofold axis co-exist in two conformations - (1) the usual inactive apo form and (2) the active ligand bound form, thus violating the symmetry of the dimer.

## Results

### Purification and Oligomeric Status of Mtb-BirA

**Figure 1:**
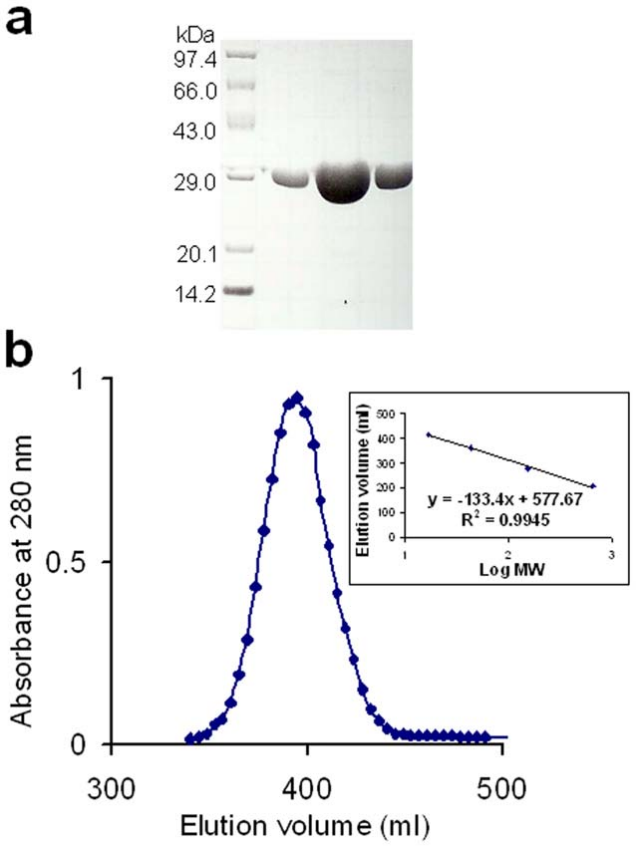
Purification and oligomeric status of Mtb-BirA. (**a**) Analysis of the purified recombinant Mtb-BirA by SDS-PAGE on a 12.5% gel. Molecular-mass protein standards are also indicated. (**b**) Gel-filtration chromatography profile of the affinity purified Mtb-BirA for subunit determination. Calibration curve for the packed Sephadex G-200 column (2.5 cm×90 cm) obtained by plotting the logarithms of the molecular weight (log MW) of the standards (x axis) versus the elution volumes (y axis) is shown as an inset.

Recombinant Mtb-BirA was overexpressed in *E. coli* and purified by Strep-Tactin affinity chromatography (Figure 1a). The subunit composition of the purified protein was determined by gel filtration chromatography using standard protein markers (Figure 1b). Based on the chromatographic profile, the Mtb-BirA corresponds to a monomer in solution as reported by Purushothaman *et al*. [7].

### Structures of Dehydrated and Hydrated Mtb-BirA Crystals

**Figure 2:**
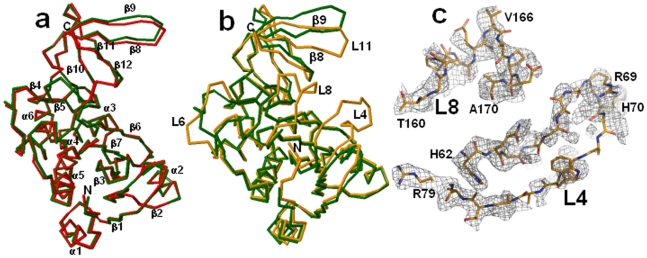
Structure of dehydrated vs hydrated Mtb-BirA. (**a**) Superimposition of backbones of subunit A of 2cgh (green) and hMtb-BirA (red) by using RAPIDO indicates similar structures. The secondary structures are indicated. (**b**) Superimposition of backbones of subunit A of 2cgh (green) and dhMtb-BirA (orange) by using RAPIDO displays shifts in L6 as well as in the β8-L11-β9 region and the appearance of two missing loop regions L4 and L8 and 5 N-terminal residues in the later structure. (**c**) Sigma weighed 2 *F_o_–F_c_* electron density maps (gray mesh) contoured at 0.7σ around the modeled L8 and L4 loops (represented as sticks in atom type colors).

**Figure 3:**
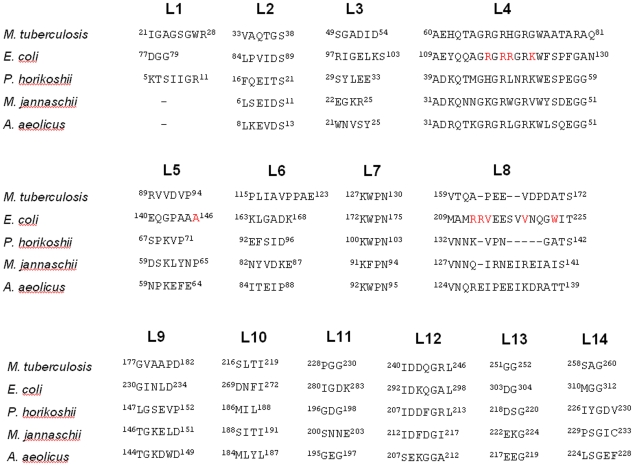
Loop sequence diversity among BirAs. Loops and turns in Mtb-BirA and equivalent sequences in homologues (the first and the last residue numbers are indicated before and after each loop) are represented. The important residues identified by mutational studies in EcBirA are marked in red.

Structures of dehydrated (dhMtb-BirA) and hydrated (hMtb-BirA) Mtb-BirA, were determined at 2.69 Å and 2.8 Å, respectively, using crystals grown under similar conditions. The hydrated structure contains 44% solvent content similar to that of Mtb-BirA structure (45.4%) solved at a resolution of 1.8 Å (PDB entry: 2cgh). The asymmetric unit for both hydrated crystal forms (2cgh and hMtb-BirA) includes two monomers in the asymmetric unit. Both the crystal structures are quite similar as indicated by the root-mean-square difference (rmsd) of 0.69 Å for 480 equivalent Cα atom pairs. The backbone structures of subunits A as superimposed with *RAPIDO* do not display any significant difference between the two structures either (Figure 2a). The Mtb-BirA molecule is composed of two domains; N-terminal domain 1 comprises of 7 β-strands (β1: 29–32, β2: 55–59, β3: 82–88, β4: 124–126, β5: 131–133, β6: 137–147 and β7: 150–158) and 6 α-helices (α1: 13–20, α2: 39–48, α3: 95–114, α4: 173–176, α5: 183–203, α6: 206–215), while C-terminal domain 2 is a SH3 domain with 5 strands forming an antiparallel β-sheet (β8: 220–227, β9: 231–240, β10: 246–250, β11: 253–257 and β12: 261–264). The major loops (Figure 3) in domain 1 are: L1 (21–28), L2 (33–38), L3 (49–54), L4 (60–81), L5 (89–94), L6 (115–123), L7 (127–130), L8 (159–172) & L9 (177–182) and in domain 2 are: L11 (228–230), L12 (241–245), L13 (251–252) & L14 (258–260). The two domains are linked together with loop L10 (216–219) that connects helix α6 of domain 1 and β8 strand of domain 2. The 7 N-terminus residues and two loop regions between residues 65–76 (L4) and 162–169 (L8) are not there in both subunits of high (1.8 Å, 2cgh) and low-resolution structures (2.8 Å, hMtb-BirA). The disordered loops are undetectable in other BirA structures (1bia, 1wq7 and 3fjp) as well and are associated with the conformational changes upon biotinyl-5′-AMP binding [12]–[14].

**Figure 4:**
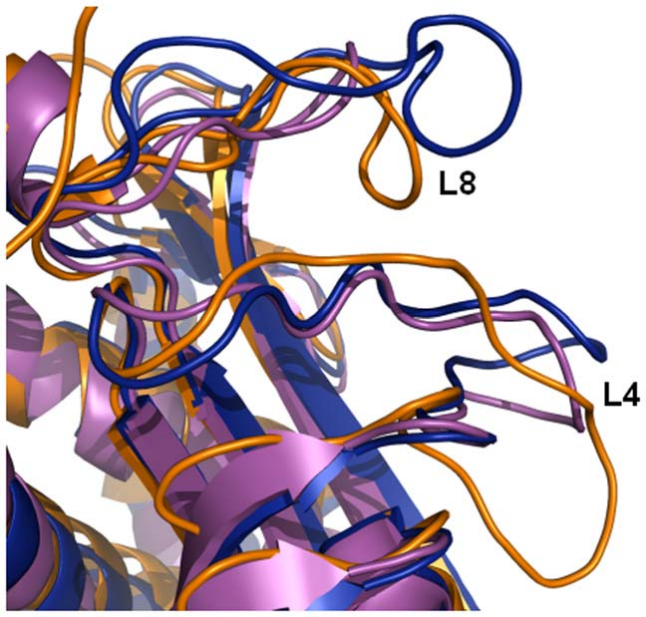
Dehydration induced structural ordering of L4 and L8 loops corresponds to active conformations. Superimposition of cartoon representation of subunit A of dhMtb-BirA (orange) onto EcBirA (2ewn, blue) and PhBirA (1wqw, magenta). Dehydration induced structural appearance of L4 and L8 loops in dhMtb-BirA coincides with the active conformation of these loops on ligand-binding.

The asymmetric unit of dehydrated crystal form containing two molecules is more compact and densely packed with a low solvent content of 28% compared to the hydrated crystal forms. Analysis of the two crystal forms shows the core regions to be identical, but some significant conformational plasticity is clearly visible among the loops (Figure 2b) leading to a relatively higher rmsd of 2.03 Å for 454 equivalent Cα positions between 2cgh and dhMtb-BirA. Large shift of ∼3.5 Å in the flexible loop L6 as well as in the β8-L11-β9 region is an apparent consequence of crystal dehydration. Additionally, the most interesting feature is the appearance of two missing loop regions L4 and L8 and 5 N-terminal residues in subunit A of the dhMtb-BirA (Figure 2b). The two missing loops L4 and L8 were built following the visible electron density at half and full occupancy, respectively. The electron density for loop L8 leaves little doubt as to the position of the atoms (Figure 2c) and though electron density for loop L4 is not complete, apex of this loop constituting residues R69 and H70 has a well-defined density (Figure 2c). Although L4 displays broken electron density associated with it, the original weak positive density, without any model built into it, improved markedly after loop building and did not exhibit any negative density, increasing our confidence in the modeled loop. Furthermore, it is interesting that position of these density guided built loops (subunit A) turns out to be very close to the ligand bound active loops (Figure 4) reported in EcBirA (2ewn) and PhBirA (1wqw) and hence the subunit A of dhMtb-BirA corresponds to the active form of the enzyme.

### Water-Mediated Closed Structure of Loop L6

**Figure 5:**
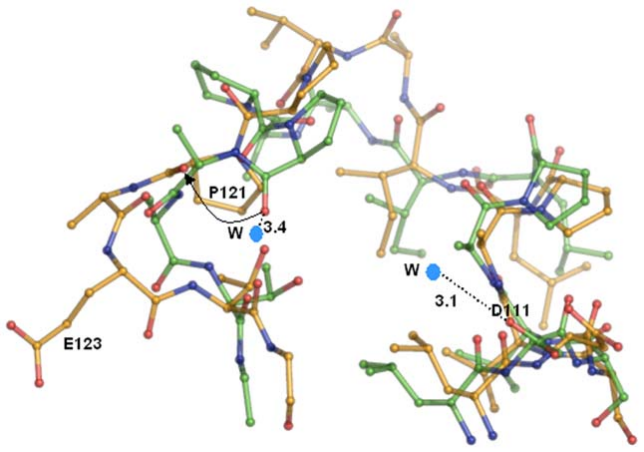
Water-mediated closed structure of L6 loop. Conformation of loop L6 in hMtb-BirA (green) and dhMtb-BirA (yellow) when rest of the subunit A of two structures is superimposed. The two water molecules associated with D111 and P121 in hMtb-BirA are marked W and respective distances are labeled. An arrow depicts the outward orientation shift of P121 main chain oxygen in the absence of water in dhMtb-BirA.

Two water molecules can be seen associated with residues P121 and D111 at the distance of 3.4 Å and 3.1 Å, respectively, in loop L6 of hMtb-BirA, whereas, these residues are no longer hydrated in dhMtb-BirA (Figure 5). We have used the hydrated structure instead of high-resolution 2cgh for water related comparisons as similar crystallization conditions and structure resolutions for hMtb-BirA and dhMtb-BirA make the analysis more valid. The presence of water molecules in the hydrated structure keeps this loop in a closed-compact conformation. Loss of water molecules and hence associated interactions has resulted in the opening out of this loop in the dehydrated structure. Further, in hMtb-BirA (as well as 2cgh), the side chains of the residue E123 and neighboring residues R135 & R215 are not defined beyond Cβ but they are well defined in the subunit A of dhMtb-BirA, a consequence probably of outward movement of loop L6.

### Asymmetric Dimer in dhMtb-BirA

**Figure 6:**
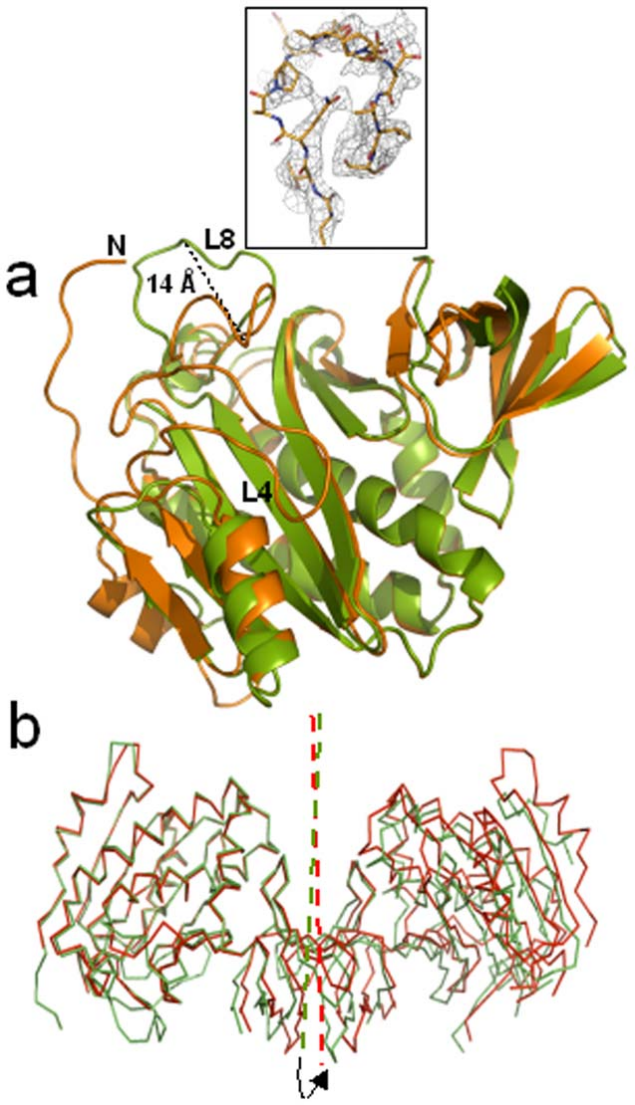
The asymmetric dimer. (**a**) Superposition of cartoon representations of dhMtb-BirA subunit A (orange) and subunit B (green) exhibiting the structural differences in two subunits. Seven N-terminal residues and loop L4 are disordered in subunit B and have not been built. Maximum conformational differences in the two subunits are displayed in loop L8 with 14 Å shift measured at the apex of the loop. Inset shows the sigma weighed 2 *F_o_–F_c_* electron density map (gray mesh) contoured at 0.7σ around the L8 loop in subunit B of dhMtb-BirA (represented as sticks in atom type colors). (**b**) The dimeric molecule in the asymmetric unit of dhMtb-BirA (green) and hMtb-BirA (red) are shown after superposition of subunit A. The arrow indicates an anticlockwise rotation (7°) of the twofold axis that relates to the two monomers (indicated in the same color) required for subunit B of dhMtb-BirA to superpose on the corresponding subunit of hMtb-BirA.

The two subunits of dhMtb-BirA have structural differences in their loops and defy the expected two fold symmetry resulting in an asymmetric dimer where, subunit A can be considered a representation of the active ligand bound conformation of BirA and subunit B is more an icon of apo BirA with disordered loops. The rmsds between the two subunits of dhMtb-BirA are 0.887 Å for 198 Cα equivalent atoms as opposed to the rmsds of 0.578 Å for 208 Cα pairs of the hMtb-BirA subunits. The maximum deviation of ∼14 Å appears to be in L8 loop, which is adenosine binding loop (Figure 6a) and may correspond to the conformational shift this loop undergoes upon biotin or biotinyl-5′-AMP binding. A noncrystallographic dyad exists between the two subunits in both hMtb-BirA and dhMtb-BirA structures. Although the mode of dimerization is same, small differences are observed in the mutual orientation of the subunits in the dimer after superposing the A subunits of two structures (Figure 6b). The angle of rotation necessary for superposing the B subunits turns out to be nearly 7°.

### Dimer Interface Variations among BirAs

**Figure 7:**
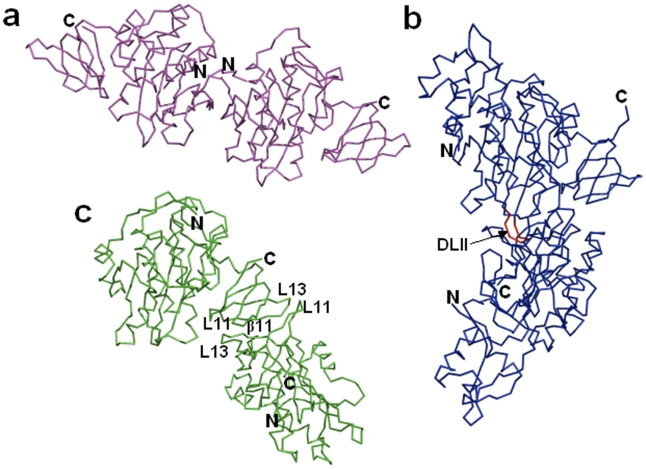
Structural conservation and dimer interface in BirA. (**a**) N-terminal dimer interface in apo PhBirA (pdb code:1wq7). (**b**) Ligand induced dimer interface involving DLI and DLII loops in EcBirA (pdb code:2ewn). The extended DLII loop in *E. coli* (shown in red color and depicted by arrow) is known to be involved in the ligand induced dimerization and is shorter in the other two variants of BirA. (**c**) C-terminal dimer interface in apo dhMtb-BirA. The structural elements of each monomer at the dimer interface are labeled.

The pseudo-2-fold symmetric interface shared by two monomers of the asymmetric unit in both hMtb-BirA and dhMtb-BirA is ascribed to the C-terminal domain involving β11 strands and loops L11 (between β8–β9) and L13 (between β10–β11) of two monomers (Figure 7c). The residues E226, E231, V233, D241, V250, V255, V256 and S258 of chain B interact with the interface residues L227, P228, V233, V234, R245, R253, T254 and V256 of chain A. This dimeric interface of dhMtb-BirA is different from Ec and Ph BirAs. The ligand induced dimeric interface in the crystal structure of EcBirA (Figure 7b) involves a β strand (corresponding to β6 in Mtb-BirA) and the loops DLI (L5 in Mtb-BirA) and DLII (corresponding loop between β6–β7 is absent in Mtb and Ph BirAs). On the other hand, dimerization in PhBirA crystals occurs through N-terminal β strands of two monomers (Figure 7a).

## Discussion

Crystals of Mtb-BirA used in the present investigation were grown in the conditions (12–16% PEG 4000 in 0.1 M HEPES pH 7.5) different to those used for growing crystals in case of 2cgh. Yet, both the dehydrated (dhMtb-BirA) and hydrated (hMtb-BirA) crystals belonged to the same space group as 2cgh. However, post-crystallization soaking of crystals in cryoprotectants followed by flash freezing resulted in a dehydrated structure with changes in unit cell volume and low solvent content [18].

Structural changes as a result of crystal dehydration have been observed and studied extensively in many proteins like lysozyme and bovine pancreatic ribonuclease A [19]–[22]. Water mediates an essential role in maintaining structure, stability and functionality of biological macromolecules not only in solution but also in the crystalline form. A systematic reduction in the solvent content of protein crystals has demonstrated that reduction in the quantity of surrounding water affects the hydration shell of proteins, shrinks the crystal lattice and improves the crystal packing and diffraction resolution [23]–[25]. Moreover, as solvent reduces and additional packing interactions are introduced, possibility for flexible loops to adopt local structures increases and in the process kinetically favorable conformations that occur during the protein action get trapped [20], [25], [26].

This work is yet another example of a link between hydration and plasticity in proteins. As stated earlier, in all known apo BirA structures, the loops L4 and L8 are disordered. This is true for normally hydrated Mtb-BirA (2cgh as well as our hMtb-BirA) structure also. However, transfer of crystals to paraffin and paratone-N oil (cryoprotectants) before collecting data at 120 K resulted in a partially dehydrated structure. It is quite possible that oil absorbed water and dehydrated the crystal during the period it was dipped in the oil. Fortuitously, the structure solved from the dehydrated crystal exhibited electron density for the otherwise flexible L4 and L8 loops allowing model building of these loops (Figure 2c). This is the first structural example of these flexible loops being visible in the ligand free state of BirA. The overall changes in the subunit A of dhMtb-BirA structure appear to mimic those that arise during substrate binding (Figure 4) and corroborate well with the established relationship between hydration, mobility and enzyme action [20].

The ordered loops L4 and L8 in the structure of dehydrated apo BirA form an open, large and spacious biotin-binding pocket. The two different conformations for L8 in the two subunits of dhMtb-BirA exhibit a shift of 14 Å (Figure 6a). The available evidence from crystal structures of apo and ligand bound BirA's lead to the inference that L8 structure in subunit A represents the active conformation, while that in subunit B (with more peripheral placement) may mimic the inactive open state. As the initial structure of this L8 loop has not been observed before, characterizing the L8 structure (subunit B) from dhMtb-BirA as ligand free form may appear to be assumptive to begin with, however, all other observations point towards subunit B being a snapshot of apo BirA leaving little doubt that for the first time we have the molecular structure of the L8 loop available to us. The biotin-binding pocket is conserved in BirA variants from different organisms and is lined with residues from L4 (G66, G68 and W74) and β7 (G154 and G156). The conserved glycine and tryptophan residues are required to cover the biotin ring and AMP moiety, respectively. Despite the highly conserved pocket, variable dissociation constants for biotin have been documented for EcBirA (4.5×10^−8^ M), *Aquifex aeolicus* (Aa) BirA (3.5×10^−6^ M) and Mtb-BirA (∼1×10^−6^ M) [7], [14], [27]. Similarly, a lower affinity for Mtb-BirA:biotinyl-5′-AMP and Mtb-BirA:ATP interactions compared to *E. coli* homologue has also been reported. In addition, a gain of −4 kcal/mol of Gibbs free energy was observed on binding of EcBirA to biotinyl-5′-AMP over biotin, contrary to the similar gain of Gibbs free energies for binding of these two ligands to Mtb-BirA [7]. The free energy of binding between protein and its ligand includes an electrostatic and a hydrophobic component and analysis of these components provides an answer for these differential affinities. Crystal structure of adenylate bound *E. coli* enzyme reveals that the ABL loop (L8 in Mtb-BirA) folds by forming a hydrophobic cluster involving side chains of V214, V219 and W223 around the adenine base [12]. Single amino acid replacement of these hydrophobic residues with Ala in EcBirA exhibited lower affinities for the adenylate ligand as compared to the wild type [16]. This hydrophobic cluster is critical not only for ATP binding but also for the ligand induced disorder to order transition of this loop. The loop L8 is shorter in Mtb-BirA and is devoid of these hydrophobic residues (Figure 3) and hence, maybe responsible for the lower affinities for ATP and biotinyl-5′-AMP ligands as compared to EcBirA.

Another intriguing feature that emerges from the structure of Mtb-BirA relates to the loop L6. This loop is extended, more proline rich and hydrophobic as compared to the corresponding loop in other counterparts (Figure 3) and protrudes out from rest of the protein. Further opening of this loop in the dhMtb-BirA (Figure 5) akin to water mediated loop closure in β-lactoglobulin on dehydration [26] provides an image of changes that might occur during protein action. It has been shown that exposed positioning of the proline rich loops mediates interactions with other proteins [28]. Non-repetitive proline rich regions acting as a ‘sticky arm’ are known to bind rapidly and reversibly to SH3 domains through hydrophobic interactions. Coincidentally, L6 is placed adjacent to the C-terminal domain of the Mtb-BirA that bears similarity to SH3 domain, indicating interaction between these components of the protein. On the other hand, as proline is an unusual amino acid with restricted conformation, its multiple presence in this loop may provide an extended surface with limited mobility, suggesting a ‘rigid hinge or a linker’ role for L6. In Mtb-BirA, L6 is connecting α3 and β4 where the later is followed by a conserved loop L7 (^127^KWPN^130^). This L7 loop is crucial in the placement of BCCP domain of ACCases and PCAases for biotin transfer. Therefore, a more rigid L6 linker may contribute to the spatial positioning of recognition elements involved in positioning/transfer of biotin to BCCP domain. Though these postulations need to be validated, it does appear that this unique region present only in the Mtb complex may be critical for BirA's function and specificity.

Comparisons of oligomeric states of BirAs, both in solution and crystals, from *M. tuberculosis*, *E. coli, P. horikoshii*, *Methanococcus jannaschii* and *A. aeolicus*, have revealed an immense structural and functional diversity (Table1). Though, Mtb-BirA packs as a dimer in the asymmetric unit of the crystal, it exists as a monomer in the unliganded as well as liganded forms in solution [7], contrary to Ec and Ph BirAs. Thermodynamic and enzyme kinetic studies have clearly established the role of DLII loop and other structural elements in promoting the ligand induced dimerization in EcBirA [17]. This loop consists of a β turn of two residues (Q148–P149) in Mtb and hence, shortening of this loop element maybe correlated with the monomeric functional state of the enzyme in this organism. The presence/absence of loops has been known to play a role in enabling/disabling homo-oligomerization of proteins [29]. PhBirA that lacks this DLII loop is still present in solution as a dimer in both apo and holo states albeit through a different dimer-interface. In addition, only few of the residues (R118, A146 and K122) important for homodimerization in EcBirA (R118, R119, A146, K122, R212, V214, V219 and W223 marked red in Figure 3) are conserved in Mtb and Ph BirAs further supporting the oligomeric diversity. It appears that this dimerization event is not a requirement for the enzymatic activity, but is required for repressor activity in EcBirA [15], [17] and for thermal stability as proposed for PhBirA and other hyperthermophiles [11], [30]. Mtb-BirA has neither N-terminal HTH DNA binding motif for repressor activity nor a requirement for thermostable character and hence, formation of a homodimeric state may not be advantageous in its environment. Moreover, it is crucial for some proteins to maintain their monomeric state for various reasons such as rapid diffusion and stability at low concentrations [31].

**Figure 8:**
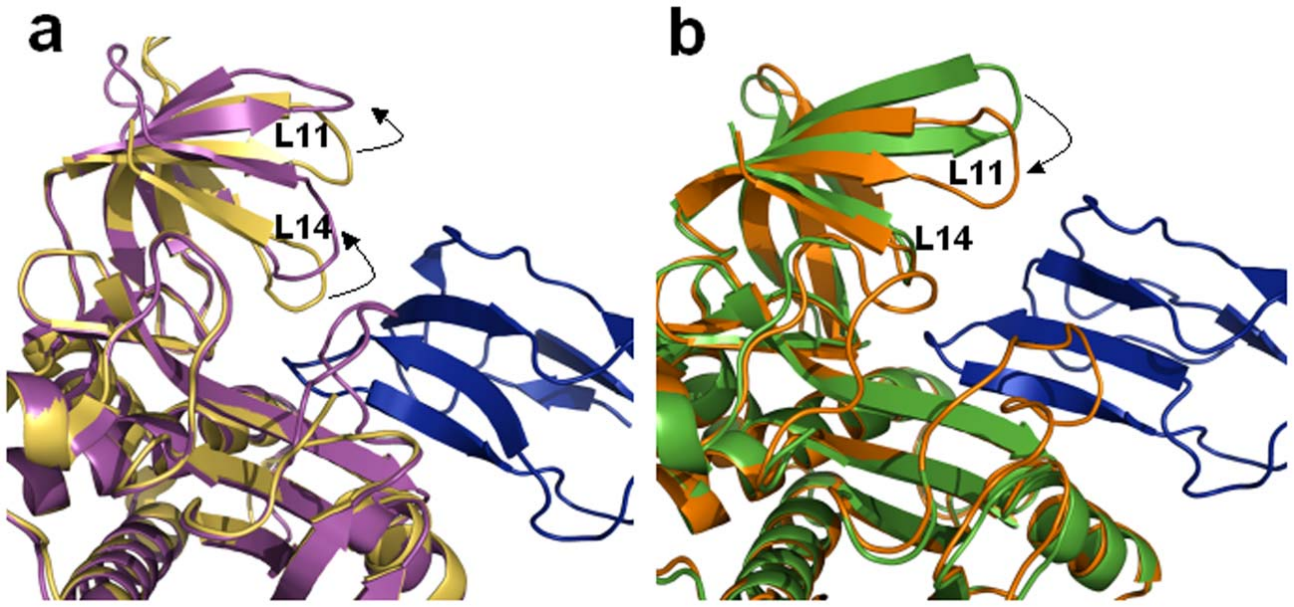
BirA-BCCP complex. (**a**) Structural superposition of cartoon representations of subunits A of apo (yellow) and BCCP complexed (magenta) PhBirA illustrating the open/close movement (marked by an arrow) of L14 loop to regulate the entry/exit of BCCP. (**b**) Similar superposition of hMtb-BirA (green) and dhMtb-BirA (orange) representing apo and active forms, respectively, demonstrate no such movement in L14 loop. Contrary to the movement of C-terminal loops in PhBirA, the loop L11 in Mtb-BirA moves inwards (marked by an arrow) on BCCP binding. BCCP molecule in both figures is shown as blue cartoon.

Plasticity around the active site of PhBirA has been seen to assist in complex formation with its substrate BCCP [13]. In addition, superposition of apo and holo PhBirA structures exhibits maximum variations in the C-terminal domain, especially in L11 and L14 loops (Figure 8a). It has been proposed that the loop L14 involving the residue Y227 (*P. horikoshii* sequence in Figure 3) undergoes an open/close motion to regulate the movements of ligands. In the active complex, L11 and L14 loops shift outwards to place BCCP at the active site. However, the docking of modeled BCCP domain of Mtb ACCA3 in the hMtb-BirA and dhMtb-BirA, representing free and ligand bound states of Mtb-BirA, respectively, reveals contrary movements of these loops (Figure 8b). The loop L14 in Mtb structure is only three residues long and is devoid of the tyrosine residue Y227 (Figure 3). Moreover, the absence of any significant structural change in this very short loop negates its possible involvement during substrate binding. But the closing in of L11 loop in the Mtb-BirA BCCP complex put forward the notion that the ligand placement role of L14 in phBirA is carried out by L11 in Mtb. Interestingly, this loop does not appear to be involved in any gated mechanism for the entry/exit of BCCP substrate thus providing a constitutive access to the ligand. This Mtb specific behavior of C-terminal domain justifies the need for accommodating different BCCP domains [32] and biotinylation turnover necessary for the biosynthesis of unique fatty acids in Mtb under varying environmental conditions.

The dehydrated crystal structure of apo Mtb-BirA provides another example of water-mediated transformations emulating protein in action. BirA, an elegant enzyme that carries out multi-step chemical reaction, has conserved core structure and multiple loops participating in protein folding, ligand-binding and homo/hetro dimerization. Dictated by the need of an organism, these loops have evolved under constraints, weaker than those for a protein core yet strong enough to preserve the overall structure, for fulfilling the specific requirement of the organism. Understanding and correlating those critical variables with an environment that BirA is operating in, may be the key to successfully decipher the working of this complex enzyme. Final confirmation relating these dehydration-induced changes to protein in action awaits the structure solution of BirA with its ligands.

## Methods

### Protein Expression, Purification, Crystallization and Subunit Composition

The gene encoding BirA (Rv3279c) was PCR amplified with C-terminus Strep-tag, cloned in pASK-IBA43plus (IBA) vector and expressed in *E. coli* BL21 (DE3) cells after induction with 1 mM IPTG at 37°C as previously reported [18]. The protein was purified by using Strep-Tactin resin (IBA), concentrated to ∼5 mg/ml and crystallized at 293 K in 12–16% (w/v) of PEG 4000 or PEG 8000 in 0.1 M HEPES pH 7.5 by hanging drop method. Subunit composition of the protein in solution was analyzed by gel filtration chromatography. A 500 ml column packed with Sephadex G-200 resin (pre-equilibrated with 0.1 M Tris-HCl buffer pH 8.0 containing 0.1 M NaCl) was used for the estimation of molecular mass of the affinity-purified protein.

### Data Collection, Processing and Molecular Replacement

Two data sets were collected on these crystals (i) at room temperature (hMtb-BirA) with synchrotron radiation source of X13 beamline (EMBL, Hamburg) to 2.8 Å resolution and (ii) at cryogenic temperature (dhMtb-BirA) with a MAR345 dtb detector (MAR Research) using a home source rotating-anode generator (Rigaku) to 2.69 Å. For the low temperature data, crystals were quick-dipped into 1∶1 mixture of Paraffin and Paratone-N oil for cryo-protection and immediately flash frozen in gas stream of liquid nitrogen prior to data collection. Data for hMtb-BitA under cryo conditions could not be collected, as usual cryoprotectants lowered the resolution and exhibited a diffused pattern [18]. The data for 2cgh structure (solvent content of 45.4%) has been collected under cryo conditions and hence is used as a reference for hMtb-BirA under cryo conditions. The data collection, processing statistics and molecular replacement solution for both the data sets is summarized in Table 2. Crystals of hMtb-BirA exhibited a solvent content of 44% comparable to the reported hydrated BirAs (Table 1) as opposed to dhMtb-BirA that had an unusually lower solvent content of 28%.

**Table 2:** Data collection and refinement statistics for hMtb-BirA (3l2z) and dhMtb-BirA (3l1a).

Statistics	3l2z	3l1a
Diffraction data		
Space group	P2_1_2_1_2_1_	P2_1_2_1_2_1_
Unit-cell (Å)	a = 79.7,b = 62.8,c = 105.8	a = 60.1,b = 64.0,c = 103.6
Temperature (K)	295	120
Wavelength (Å)	0.8088	1.5418
Crystal-detector distance (mm)	170	100
Resolution limit (Å)	15-2.8 (2.9-2.8)	24-2.69 (2.82-2.69)
Exposure time per image (s)	4	300
No. of observed reflections	89516	44885
No. of Unique reflections	15831	13250
Completeness (%)	99.4 (99.6)	98.7 (96.8)
Average redundancy	5.7 (5.8)	3.2 (3.25)
Mean *I/σ (I)*	17.2 (2.2)	4.3 (2.1)
† *R*_merge_ (%)	9.8 (46.2)	8.7 (34.9)
No. of molecules in ASU	2	2
Matthews coefficient (Å^3^ Da^−1^)	2.2	1.7
Solvent content (%)	44	28
Refinement and model quality		
Resolution (Å)	14-2.8 (2.89-2.80)	23.99-2.69 (2.81-2.69)
No. of reflections used	13373 (1188)	11423 (1245)
Completeness (%)	99.3 (100)	99.2 (99)
‡R_work_/§R_free_ (%)	17/23 (24.5/33.5)	23/31 (26.6/37.4)
rms deviation bond lengths (Å)	0.007	0.009
rms deviation bond angles (°)	1.036	1.350
Average B factor (Å^2^)	45.7	43.6
Number of protein/solvent atoms	3535/26	3579/31
Ramachandran validation		
Residues in favoured regions (%)	97.2	90.8
Residues in allowed regions (%)	2.4	4.8
Residues in disallowed regions (%)	0.4	4.4

Values in parentheses are for the highest resolution shell.

† *R*_merge_ = Σ*_hkl_*Σ*_i_* |*I_hkl_* −<*I_hkl_*>|/Σ*_hkl_*Σ*_i_I_hkl_*, where *I_hkl_* is the intensity of an individual measurement of the reflection with Miller indices *h*, *k* and *l* and <*I_hkl_*> is the mean intensity of redundant measurements of that reflection.

‡ *R*_work_ = Σ*_hkl_* |*Fo_(hkl)_*−*Fc_(hkl)_*|/Σ*_hkl_* |*Fo_(hkl)_*|, where *Fo* and *Fc* are observed and calculated structure factors, respectively.

§ *R*_free_ calculated for a randomly selected subset of reflections (10%) that were omitted during the refinement.

### Refinement

The structures obtained were refined with the PHENIX refinement package phenix.refine [33] where intermittent model building was performed by using COOT [34]. Refinement statistics are summarized in Table 2. Water molecules were positioned into well-defined positive (*F_o_ –F_c_*) difference densities with a lower cutoff of 3σ, if they participated in hydrogen bonds to either the protein or to well established water molecules. They were removed, if their isotropic temperature B-factor refined to a value exceeding 60 Å^2^. Unexpectedly, inspection of the electron density map of dehydrated structure indicated weak density for the loop segments corresponding to residues 65–76 and 162–169 in chain A of the asymmetric unit. This electron density guided manual rebuilding, allowed to trace these loops with confidence in main chain position. The geometrical quality of the model was assessed with PROCHECK [35]. The Ramachandran plot shows that ∼96% of the residues fall into the favorable/allowed regions. The outliers are either supported by the density or are present in the aforementioned loops.

### Homology Modeling of BCCP Domain of Mtb-ACCA3

**Figure 9:**
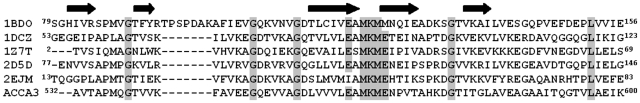
Structure-based multiple sequence alignment of BCCP domain of Mtb-ACCA3. The structure-based sequence alignment of BCCP domain of ACCA3 was generated by MODELLER 9v6 with known structures from different sources as identified by their PDB codes (*E. coli* BCCP:1bdo, *P. shermanii* transcarboxylase:1dcz, *B. subtilis* BCCP:1z7t, *P. horikoshii* BCCP:2d5d and human ACC:2ejm). The first and the last residue numbers are indicated before and after each sequence. The topological positions of β strands, as defined in the known structures of BCCPs are shown on the top of the aligned sequences. Identical residues including MKM motif are highlighted in gray boxes.

BCCP domain of ACCA3 (Rv3285) protein of Mtb was modeled by MODELLER 9v6 [36] based on its homology with multiple templates (Figure 9) of known NMR or crystal structures of BCCP or BCCP containing proteins (*E. coli* BCCP:1bdo, *Propionibacterium shermanii* transcarboxylase:1dcz, *Bacillus subtilis* BCCP:1z7t, *P. horikoshii* BCCP:2d5d and Human acetyl Co-A carboxylase:2ejm). The model with the minimum DOPE score was selected as the best model and the stereochemical quality of the model was evaluated and confirmed with PROCHECK.

### Modeling Mtb-BirA-BCCP Complex

The modeled BCCP domain was docked manually onto the subunit A of dhMtb-BirA by using the crystal structure of BirA and BCCP complex from *P. horikoshii* OT3 (2ejf) as a template. To fix the orientation of the ligand to the receptor, dhMtb-BirA and modeled BCCP domain were structurally superimposed on 2ejf: Chain A and 2ejf: Chain C representing homologous BirA and BCCP, respectively, from *P. horikoshii*. This model was further minimized in Discovery Studio 2.1 (using CHARMM forcefield) to improve the energies and eliminate clashes.

### Coordinates

Coordinates and structure factors for hMtb-BirA and dhMtb-BirA are deposited in the RCSB Protein Data Bank (www.rcsb.org) under accession code 3l2z and 3l1a, respectively.

## References

[1] Parrish NM, Houston T, Jones PB, Townsend C, Dick JD (2001). In vitro activity of a novel antimycobacterial compound, N-octanesulfonylacetamide, and its effects on lipid and mycolic acid synthesis.. Antimicrob Agents Chemother.

[2] Bhatt A, Molle V, Besra GS, Jacobs WR, Kremer L (2007). The *Mycobacterium tuberculosis* FAS-II condensing enzymes: their role in mycolic acid biosynthesis, acid-fastness, pathogenesis and in future drug development.. Mol Microbiol.

[3] Chapman-Smith A, Cronan JE (1999). The enzymatic biotinylation of proteins: a post-translational modification of exceptional specificity.. Trends Biochem Sci.

[4] Beckett D (2007). Biotin sensing: universal influence of biotin status on transcription.. Annu Rev Genet.

[5] Beckett D (2009). Biotin sensing at the molecular level.. J Nutr.

[6] Polyak SW, Chapman-Smith A, Mulhern TD, Cronan JE, Wallace JC (2001). Mutational analysis of protein substrate presentation in the post-translational attachment of biotin to biotin domains.. J Biol Chem.

[7] Purushothaman S, Gupta G, Srivastava R, Ramu VG, Surolia A (2008). Ligand specificity of group I biotin protein ligase of *Mycobacterium tuberculosis*.. PLoS One.

[8] Mukhopadhyay B, Purwantini E, Kreder CL, Wolfe RS (2001). Oxaloacetate synthesis in the methanarchaeon *Methanosarcina barkeri*: pyruvate carboxylase genes and a putative Escherichia coli-type bifunctional biotin protein ligase gene (bpl/birA) exhibit a unique organization.. J Bacteriol.

[9] Rodionov DA, Mironov AA, Gelfand MS (2002). Conservation of the biotin regulon and the BirA regulatory signal in Eubacteria and Archaea.. Genome Res.

[10] Wilson KP, Shewchuk LM, Brennan RG, Otsuka AJ, Matthews BW (1992). *Escherichia coli* biotin holoenzyme synthetase/bio repressor crystal structure delineates the biotin- and DNA-binding domains.. Proc Natl Acad Sci U S A.

[11] Bagautdinov B, Kuroishi C, Sugahara M, Kunishima N (2005). Crystal structures of biotin protein ligase from *Pyrococcus horikoshii* OT3 and its complexes: structural basis of biotin activation.. J Mol Biol.

[12] Wood ZA, Weaver LH, Brown PH, Beckett D, Matthews BW (2006). Co-repressor induced order and biotin repressor dimerization: a case for divergent followed by convergent evolution.. J Mol Biol.

[13] Bagautdinov B, Matsuura Y, Bagautdinova S, Kunishima N (2008). Protein biotinylation visualized by a complex structure of biotin protein ligase with a substrate.. J Biol Chem.

[14] Tron CM, McNae IW, Nutley M, Clarke DJ, Cooper A (2009). Structural and functional studies of the biotin protein ligase from *Aquifex aeolicus* reveal a critical role for a conserved residue in target specificity.. J Mol Biol.

[15] Kwon K, Streaker ED, Ruparelia S, Beckett D (2000). Multiple disordered loops function in corepressor-induced dimerization of the biotin repressor.. J Mol Biol.

[16] Naganathan S, Beckett D (2007). Nucleation of an allosteric response via ligand-induced loop folding.. J Mol Biol.

[17] Zhao H, Naganathan S, Beckett D (2009). Thermodynamic and structural investigation of bispecificity in protein-protein interactions.. J Mol Biol.

[18] Gupta V, Gupta RK, Khare G, Surolia A, Salunke DM (2008). Crystallization and preliminary X-ray diffraction analysis of biotin acetyl-CoA carboxylase ligase (BirA) from *Mycobacterium tuberculosis*.. Acta Crystallogr Sect F Struct Biol Cryst Commun.

[19] Salunke DM, Veerapandian B, Kodandapani R, Vijayan M (1985). Water-mediated transformations in protein crystals.. Acta Crystallographica Section B: Structural Science.

[20] Nagendra HG, Sukumar N, Vijayan M (1998). Role of water in plasticity, stability, and action of proteins: the crystal structures of lysozyme at very low levels of hydration.. Proteins.

[21] Bell JA (1999). X-ray crystal structures of a severely desiccated protein.. Protein Sci.

[22] Harata K, Akiba T (2007). Effect of a sodium ion on the dehydration-induced phase transition of monoclinic lysozyme crystals.. Acta Crystallogr D Biol Crystallogr.

[23] Heras B, Edeling MA, Byriel KA, Jones A, Raina S (2003). Dehydration converts DsbG crystal diffraction from low to high resolution.. Structure.

[24] Kuo A, Bowler MW, Zimmer J, Antcliff JF, Doyle DA (2003). Increasing the diffraction limit and internal order of a membrane protein crystal by dehydration.. J Struct Biol.

[25] Heras B, Martin JL (2005). Post-crystallization treatments for improving diffraction quality of protein crystals.. Acta Crystallogr D Biol Crystallogr.

[26] Vijayalakshmi L, Krishna R, Sankaranarayanan R, Vijayan M (2008). An asymmetric dimer of beta-lactoglobulin in a low humidity crystal form–structural changes that accompany partial dehydration and protein action.. Proteins.

[27] Kwon K, Beckett D (2000). Function of a conserved sequence motif in biotin holoenzyme synthetases.. Protein Sci.

[28] Williamson MP (1994). The structure and function of proline-rich regions in proteins.. Biochem J.

[29] Akiva E, Itzhaki Z, Margalit H (2008). Built-in loops allow versatility in domain-domain interactions: lessons from self-interacting domains.. Proc Natl Acad Sci U S A.

[30] Dams T, Auerbach G, Bader G, Jacob U, Ploom T (2000). The crystal structure of dihydrofolate reductase from *Thermotoga maritima*: molecular features of thermostability.. J Mol Biol.

[31] Goodsell DS, Olson AJ (2000). Structural symmetry and protein function.. Annu Rev Biophys Biomol Struct.

[32] Daniel J, Oh TJ, Lee CM, Kolattukudy PE (2007). AccD6, a member of the Fas II locus, is a functional carboxyltransferase subunit of the acyl-coenzyme A carboxylase in *Mycobacterium tuberculosis*.. J Bacteriol.

[33] Afonine PV, Grosse-Kunstleve RW, Adams PD, Lunin VY, Urzhumtsev A (2007). On macromolecular refinement at subatomic resolution with interatomic scatterers.. Acta Crystallogr D Biol Crystallogr.

[34] Emsley P, Cowtan K (2004). Coot: model-building tools for molecular graphics.. Acta Crystallogr D Biol Crystallogr.

[35] Laskowski RA MacArthur MW, Moss DS, Thornton JM (1993). PROCHECK: a programme to check the stereochemical quality of protein structures.. J Appl Crystallog.

[36] Eswar N, Eramian D, Webb B, Shen MY, Sali A (2008). Protein structure modeling with MODELLER.. Methods Mol Biol.

